# Deformation, Rupture, and Morphology Hysteresis of Copolymer Nanovesicles in Uniform Shear Flow

**DOI:** 10.1021/acs.langmuir.4c04200

**Published:** 2024-12-31

**Authors:** Senyuan Liu, Radhakrishna Sureshkumar

**Affiliations:** † Department of Biomedical and Chemical Engineering and the Bioinspired Institute, Syracuse University, Syracuse, New York 13244, United States; ‡ Department of Physics, Syracuse University, Syracuse, New York 13244, United States

## Abstract

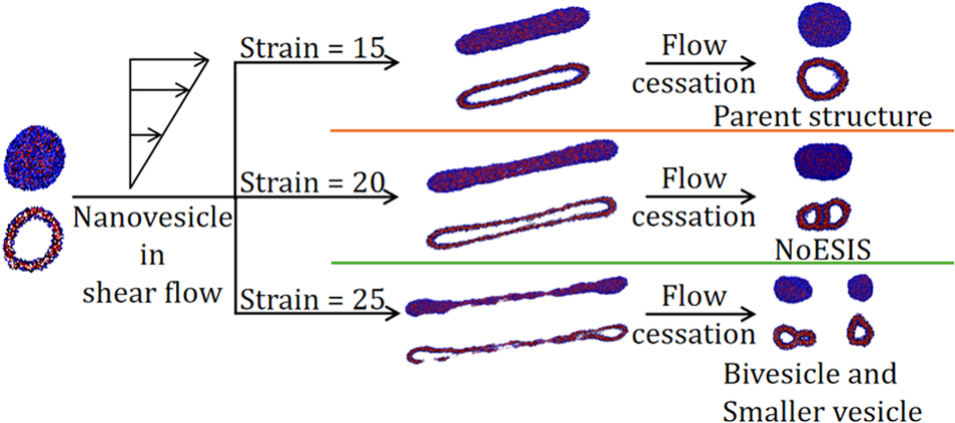

Copolymer nanovesicles are used extensively in chemical processes and biomedical applications in which they are subjected to dynamic flow environments. Flow-induced vesicle deformation, fragmentation, and reorganization modify the energetic (e.g., polymer–solvent interfacial area) and entropic (e.g., copolymer chain configuration) contributions to the solution free energy. Equilibration of a deformed morphology by flow cessation could reorganize the system into a self-assembled state, which is different from the parent structure through a local free energy minimization pathway. We perform nonequilibrium molecular dynamics simulations to investigate morphology evolution in uniform shear flow of a unilamellar nanovesicle formed by the self-assembly of amphiphilic triblock copolymers in an aqueous solution. Flow strength is characterized by the Weissenberg number *Wi*, defined as the ratio of the time scale of vesicle shape fluctuations to the inverse shear rate. For *Wi* < 10, a spherical vesicle deforms into a flow-aligned ellipsoidal bilayer executing tank-treading motion. For *Wi* > 10, pronounced variations in bilayer thickness and polymer extension manifest along the contour of the elongated vesicle, which breaks up into lamellar fragments. Below a critical strain, the deformed vesicle upon flow cessation returns to its initial spherical morphology. However, for larger strains, structure reorganization after flow stoppage results in the formation of a Novel Equilibrated Shear-Induced Structure (NoESIS) in which two vesicles are connected by a dynamic molecular bridge that can accommodate additional layers of copolymers, leading to a reduction in the polymer–solvent interface area. Mechanisms of morphology hysteresis are explored via an analysis of the thermodynamic markers.

## Introduction

Amphiphilic block copolymers (BCPs) can be engineered to produce various morphologies by altering the combinations of chemically distinct polymer segments that interact selectively with their environment, such as an aqueous or organic solvent or a polymer matrix. BCP assemblies in aqueous solutions include spherical and cylindrical micelles, wormlike micelle networks, lamellae (bilayers), star/flower-like morphologies, rings/tori, bicontinuous phases, and vesicular structures, referred to as polymersomes.^1−12^ The size and physicochemical properties of polymersomes can be controlled by varying the copolymer chain composition/length, adding secondary spacer molecules, and using microfluidics-based flow focusing techniques to produce droplets of controllable sizes.^8^ Polymersomes with sizes that range between a few and tens of nanometers, i.e., nanovesicles, can be routinely manufactured. They form the building blocks of complex nanostructures that offer significant promise in nanomedicine and cancer theranostics, an approach that integrates diagnosis (e.g., by imaging) and therapeutics,^8,11,13−15^ catalysis, and nanoreactor design,^1−4^ as well as development of biomaterials that mimic cellular structure and function.^1−8,13−17^ While traditional ionic surfactants with hydrocarbon chains can form vesicular structures, manipulation of their chemical environment is often required to facilitate vesiculation, e.g., by addition of a salt or cosurfactant, which limits their biocompatibility and biomedical applications. Moreover, nonionic copolymer vesicles offer superior stability due to enhanced hydrophobicity, as evidenced by their ultralow critical micelle concentrations compared to those of traditional surfactants.

Equilibrium self-assembly of diblock and triblock copolymers in solution has been investigated extensively by experiments, self-consistent field theory (SCFT), mesoscopic simulations, and molecular dynamics studies.^12,18−48^ Real-time visualization of the self-assembly processes in copolymer solutions is challenging due to the limitations of current imaging technologies, often leading to reliance on invasive cryogenic Transmission Electron Microscopy (cryo-TEM) for direct imaging of frozen structures or noninvasive nuclear magnetic resonance (NMR) diffusometry for indirect morphology characterization.^23,24,48^ Shape transitions in BCP systems are often interpreted using simple geometric models based on the elegant packing parameter concept.^18^ Simultaneously, experimental^12,20−22,25−28^ and molecular/mesoscopic simulation^30−47^ studies have revealed an extraordinary diversity of BCP morphologies with complex topological and interfacial characteristics. Further, theoretical understanding of the energetic and entropic driving forces underlying morphology selection has been evolving based on molecular simulations that explicitly account for solvent-mediated interactions among the copolymer chains.^19,30−32^ In comparison, the literature on nonequilibrium thermodynamics of BCP assemblies is sparse. Nanomanufacturing techniques such as extrusion, electroformation of dry polymer films, droplet phase transfer, and microfluidics manipulation typically employed in the production of polymersomes involve flow-mediated pathways of molecular assembly.^49^ Tailoring the size and membrane thickness of polymersomes requires precise control over the flow parameters informed by a quantitative understanding of flow-morphology interactions. Similarly, polymersome-based drug delivery and medical imaging are administered under complex flow environments, starting from the intravenous injection or oral ingestion of the polymersome to its passage through the circulatory system and porous intravascular tissues.

The dynamics of biomimetic lipid vesicles with diameter *D* (∼10 μm) ≫ their lipid bilayer membrane thickness δ (∼nm) in uniform shear and extensional flows have been studied extensively by experiments,^50−52^ theoretical analysis,^51,53−57^ and numerical simulations.^53,55,56,58−60^ Lipid vesicles are regarded as a model system that mimics the principal features of flow-induced deformation of mammalian cells, including human erythrocytes. Under Stokes flow conditions, three regimes of vesicle dynamics have been mapped out by Deschamps et al.^52^ in a space spanned by two dimensionless variables which depend on the viscosity of the surrounding fluid μ_s_, the ratio of the viscosity μ of the fluidic membrane to μ_s_, shear rate, an effective vesicle radius, and asphericity. Vesicle motion in these three regimes is classified as (i) tank-treading in which the vesicle deforms into a steady ellipsoidal shape with its longest axis oriented at a constant angle θ with the flow direction, (ii) tumbling in which a deformed vesicle periodically flips in the vorticity direction, and (iii) trembling which is characterized by oscillations in θ and large-amplitude shape distortions. In the tank-treading regime, the variations in θ as a function of the asphericity of the vesicle can be explained by the classical theory of Keller and Skalak that describes the shear dynamics of ellipsoidal particles.^57,60^

Several justifiable assumptions invoked in the theoretical/numerical analyses of and interpretation of experimental data for the shear flow dynamics of macroscopic vesicles could break down in the case of nanovesicles for which δ/D ∼ 1. *First*, for macrovesicles, bilayer thickness and copolymer density are assumed to be uniform during flow deformation. For nanoscopic supramolecular assemblies subjected to flow, advection of constituent molecules could lead to topological nonuniformities. For instance, a rodlike cationic surfactant micelle in an aqueous solution of surfactants and counterions, initially of uniform thickness and uniform counterion density, has been shown to break up in a sufficiently strong uniaxial extensional flow due to the advection of counterions from the midregions of the micelle toward its end-caps. The depletion of counterions from the midportion of the micellar rod enhances the electrostatic repulsion between adjacent surfactant head groups and promotes micelle scission via a pinch-off mechanism.^61^ Local curvature of ionic surfactant assemblies is greatly influenced by counterion concentration along the solvent–micelle interface since electrostatic screening facilitated by counterions reduces the splay angle between neighboring surfactant molecules. Curvature variations thus caused can have a pronounced effect on morphology transitions in such systems, e.g., from spherical to rodlike and from linear to branched structures.^62^ Zheng et al.^63^ used cryo-TEM experiments to investigate the deformation and breakup of cationic surfactant vesicles in a strong extensional flow. Based on the visualization of the transient structures resulting from vesicle rupture, they hypothesized that curvature changes induced by counterion advection are responsible for the reorganization of bilayer fragments into a network of wormlike micelles.

Although the mechanisms attributable to flow-induced counterion concentration gradients are absent in amphiphilic copolymer solutions, there is ample experimental evidence for flow-induced morphology transitions in such systems which are poorly understood. For instance, Zhang et al.^64^ observed that vigorous magnetic stirring of a solution containing spherical micelles of poly(styrene-*b*-4-vinylpyridine) in chloroform resulted in the formation of wormlike micelle networks. Chen et al.·^65^ reported that the strong extensional flow created by nanopore extrusion of a solution containing spherical micelles of polystyrene-*b*-polyisoprene copolymers in tetrahydrofuran produced cylindrical structures. A similar spherical-to-cylindrical morphology transition was reported in extensional flow by Zhu et al.^66^ for an aqueous solution of polyethylene-poly(oligo(ethylene glycol)methacrylate). Further examples of flow-morphology coupling include microfluidics-assisted manipulation of copolymer assembly to produce various structures such as cylinders, bilayers, and micelle networks,^67^ use of shear rate to as a control parameter to vary the polydispersity of copolymer vesicles,^68^ shear-induced layering of polymeric micelles,^69^ and flow-alignment of copolymeric lamellar phases.^70^ However, precise quantification of the flow field, morphology deformation, and the statistics of copolymer configurations in well-designed flow systems is required to elicit mechanistic understanding of flow–structure interactions.

*Second*, the configurations of individual copolymer chains within the bilayer can be altered by flow, as the inverse of the imposed shear rate becomes comparable to the average chain relaxation time. Unfolding and stretching of polymer chains by flow could cause local variations in the mechanical properties of the bilayer. The implications of such nanoscale physics on vesicle dynamics have not been fully investigated. *Third*, the bilayer of large vesicles is assumed to be impermeable to the surrounding fluid over the course of the experimental observations. Further, the volume and surface area of the vesicle are assumed to be conserved quantities.^52^ However, the time scale of molecular exchange processes at the nanoscale may be comparable to or smaller than the time span of the experiments. Consequently, fluctuations in the water density within and around the bilayer could influence the localized solvent-mediated forces of interactions among its constituent molecules (copolymers).

*In this work, we conduct nonequilibrium coarse-grained molecular dynamics (CGMD) simulations that account for explicit solvent-mediated interactions to elucidate the role of flow-induced nonhomogeneities in the local solvation environment, bilayer thickness, and copolymer chain configuration on vesicle dynamics, rupture, and reorganization*. The nanovesicle considered in this work results from the self-assembly of an amphiphilic BAB triblock copolymer in water. Here, the “A” and “B” denote the hydrophilic and hydrophobic segments, respectively. Based on practical relevance, the force field parameters used in our CGMD simulations are selected such that A and B mimic poly(ethylene oxide) (PEO) and polybutadiene (PB), respectively. A detailed account of the equilibrium CGMD simulations of the vesicle morphogenesis starting from a homogeneous solution of copolymers may be found in our earlier work.^30^

Flow-induced deformation of fluidic structures is typically reversible, i.e., when the imposed flow is stopped, the distorted structure returns to its equilibrium shape. This is not necessarily true in the case of self-assembled amphiphilic structures. For instance, a hysteretic behavior is observed in measured stresses during the startup (i.e., when a fluid is brought to a state of constant strain rate from rest) and cessation of uniaxial extensional flows of wormlike micelle networks.^71,72^ Similarly, experiments have shown that flow deformation can induce permanent structure transitions in aqueous solutions of rodlike cationic micelles^73−75^ or produce birefringent structures that persist for long times after flow cessation in sheared wormlike micelle solutions.^76^ Such observations point to the existence of *flow-mediated thermodynamic pathways of amphiphilic assembly which are not accessible to experimental protocols designed for equilibrium conditions*. However, these experimental findings lack clear mechanistic explanations based on theoretical analysis or numerical simulations of model systems, in which the influence of flow startup–cessation cycles with prescribed strain rates on the entropic and enthalpic markers of the system can be directly calculated. *In this work, we explore the thermodynamic topography associated with the relaxation/restructuring dynamics of deformed/fractured vesicles after flow cessation*, *revealing the existence of a novel equilibrated shear-induced structure* (NoESIS).

CGMD, dissipative particle dynamics (DPD), and SCFT provide tools for detailed real-time quantification of flow-induced morphological changes in model systems with varying degrees of detail ranging from the molecular to mesoscopic length/time scales. Such simulations, combined with NMR diffusometry^24,48^ studies that can track amphiphile motion within supramolecular assemblies to infer their morphological characteristics in the native (solution) phase, hold much potential in advancing our current understanding of flow-mediated morphology transitions.

SCFT-based simulations of an amphiphilic ABA triblock copolymer solution conducted by Cui et al.^68^ showed that the shape and polydispersity of the molecular assemblies can be controlled by varying the strength of an imposed shear flow. They introduced an ad hoc free-energy term proportional to the square of a shear rate parameter into the SCFT formulation of the problem. At relatively low shear rates, the coexistence of vesicles of disparate sizes was predicted. As the shear rate was increased, a relatively uniform solution of the smaller vesicles was observed. Further increases in the shear rate resulted in the formation of small spheres and short rods. The reduced shear rate in these simulations does not have a direct counterpart to that in experiments or to dynamic time scales of shape fluctuations. Hence, interpretation of the simulation predictions in the context of a real copolymer solution subjected to a uniform shear flow is not straightforward. Liu et al.^77^ reported a DPD simulation of a diblock copolymer solution in cylindrical Poiseuille flow. Bullet-like/leaky vesicles, spherical micelles, hamburger-like micelles, and bilayers were observed by changing the degree of confinement and dimensionless shear rate. They predicted that the rupture time of the vesicle decreased in a nonlinear fashion with an increase in the imposed shear rate. Wu et al.^78^ performed several DPD simulations to study the dynamics of coil–coil and rod–coil diblock copolymer vesicles in Poiseuille flow through a nanochannel. They observed that the mechanical stability of BCP vesicles depends strongly on the rigidity of the hydrophobic blocks. In the presence of sufficient flow inertia, the coil–coil BCP vesicles undergo a shape transition from a spherical to a bullet-like shape, eventually splitting into a smaller vesicle and an oval-shaped micelle. In contrast, the rod–coil BCP vesicles deform from a spherical to a bullet-like shape, which further transitions to a parachutelike structure before rupture. A similar inertia-induced spherical-to-bullet-like shape evolution was also predicted by Chu et al.^79^ in lipid nanovesicles in cylindrical Poiseuille flow.

In the present study, we employ nonequilibrium CGMD (NE-CGMD) simulations that explicitly model solvent-mediated intermolecular interactions to study the dynamics of a BAB copolymer vesicle in startup and cessation of uniform shear flow. Specifically, the force field parameters are selected to represent poly(ethylene oxide) and polybutadiene as the A and B blocks, respectively. Vesicular structures of PB–PEO copolymers have been investigated experimentally for applications including drug delivery^13^ and catalysis for biological reactions.^2,7^ The self-assembly pathway leading to vesicle formation in PB–PEO-PB triblock copolymer solutions has been explored in detail using equilibrium CGMD simulations in our earlier work.^30^ The Reynolds number based on the radius of the equilibrium vesicle ranges between 0.15 and 2.23. Thus, the dynamics of vesicle deformation is expected to be dominated by viscous forces. The role of additional physics, arising from flow-induced variations in bilayer thickness and copolymer chain configurations, as well as (to a lesser extent) from structure dilatation, on morphology deformation and recovery is examined. We present the simulation methodologies and data analysis techniques in Section 2, results in Section 3, and conclusions in Section 4.

## Methods

### CGMD Simulations

The force field constraints of the molecular models used in this work are based on the requirements of the MARTINI force field,^80^ which has been employed in nonequilibrium simulations in the past for amphiphilic fluids, yielding predictions of flow-induced structure modifications consistent with experimental observations.^61,81−83^ Its application to the CGMD simulation of BAB copolymer self-assembly in an aqueous solution is described in a previous publication.^30^ Additional details can be found in the Supporting Information. In the present simulations, water (modeled as a four-molecule cluster following the MARTINI prescription, represented by W), butadiene monomer (B), and ethylene oxide monomer (EO) are modeled by bead types P4, C4, and SNda, respectively.^80,84^ The vesicle is formed by the self-assembly of randomly distributed PB_5_–PEO_10_-PB_5_ copolymers in an aqueous solution. Equilibrium CGMD simulations are performed by *Gromacs*. The equilibrium system generated by an NPT simulation with periodic boundary conditions has 400,000 water beads, 40,000 antifreeze water beads (modeled by bead type WF^85^), and 3883 PB_5_–PEO_10_-PB_5_ copolymer chains of which 3871 and 12 form the vesicle membrane and a small aggregate, respectively. The initial box size of the equilibrium simulations is 40 × 40 × 40 nm. The reference temperature and pressure are 300 K and 1 bar, respectively.

**Figure 1:**
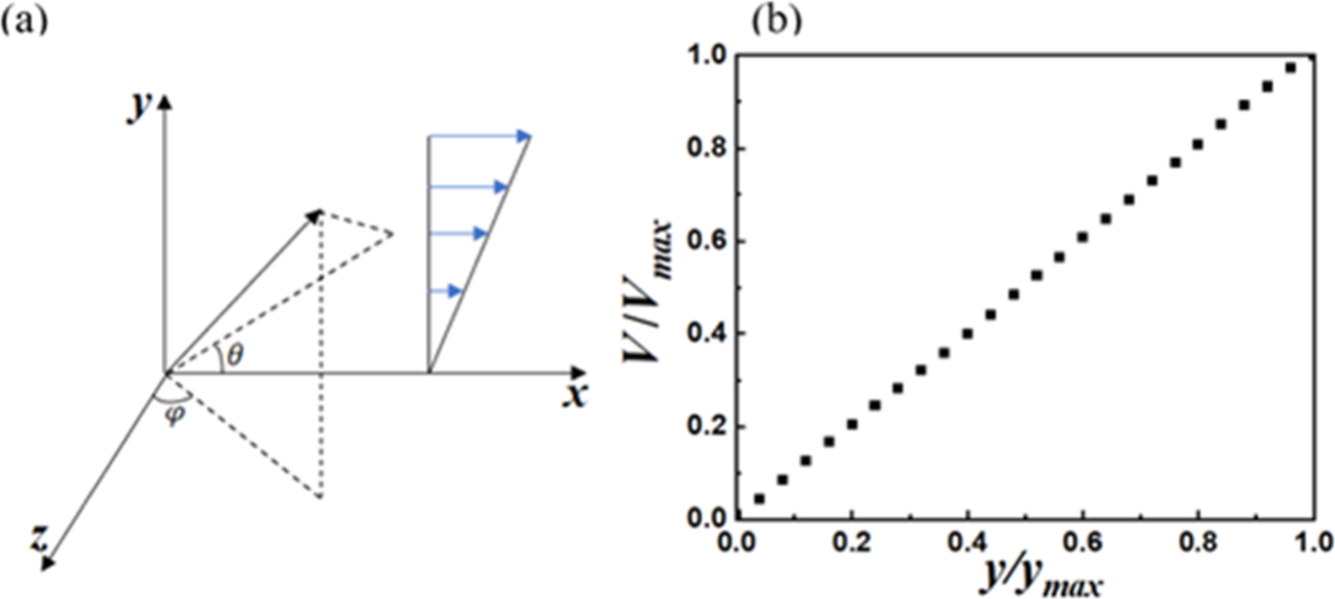
(a) Schematic of the coordinate system and uniform shear flow and (b) velocity profile realized in a typical NEMD simulation.

We use *LAMMPS* MD software to perform shear flow (NE-CGMD) simulations using the SLLOD formulation of Newton’s equations of motion. This has been shown to generate accurate particle trajectories, conserve the total momentum of the simulation system, and produce the energy dissipation required to maintain isothermal conditions.^86,87^ As shown in Figure 1a, a uniform shear flow is generated in the *x*-direction by keeping the lower boundary stationary and moving the upper boundary at a constant speed *V*. The corresponding shear rate, which is defined as the rate of change of *x*-velocity as a function of the vertical y coordinate *y*, , where *L*_*y*_ is the box size in the *y* direction. An example of the linear velocity profile realized by the simulations is shown in Figure 1b. The angle θ between the projection of the position vector of a point in space onto the *x–y* plane and the flow direction and the angle φ between its projection onto the *x*–*z* (flow–vorticity) plane and the *z*-axis are shown in Figure 1a. To account for flow-induced vesicle stretching, the box dimensions in the NE-CGMD simulations are increased to 200 × 80 × 40 nm. The additional volume thus created is filled with water and antifreeze water beads in a 10:1 ratio. The vesicle generated from the equilibrium simulations is placed into the new solvent environment, and the resulting system is equilibrated by a set of sufficiently long NPT simulations. Thus, the shear simulation system has 4,000,240 water beads, 400,000 antifreeze water, and 3871 PB_5_–PEO_10_-PB_5_ copolymer chains. This corresponds to a dilute system with a copolymer concentration of 0.122 wt %. The reference temperature is 300 K. The time step used in the NE-CGMD simulations is 5 fs, and the simulation time is 50 ns. A typical production run on a state-of-the-art 4GPU and 120 CPU cluster requires approximately 5 days. The molecular visualizations are performed using *VMD 1.9.3*. Data analysis and graphing were performed using *Matlab* 2023, *Microsoft 365*, *Visual C++ 2022*, and *Originlab* 2022.

### Data Analytics

Discrete spatiotemporal data generated from the CGMD simulations are analyzed to identify copolymer clusters. Any two beads with center-to-center separations less than the cutoff distance are regarded as part of a cluster (aggregate). Further, within any bead in each cluster, there exists at least one bead at a distance less than the cutoff value. In this study, we use 0.5 nm as the cutoff distance. This selection is based on the cutoff distance of 0.47 nm for nonbonded LJ interactions between PB and PEO monomers. We also calculate the solvent-accessible surface area (SASA), which may be interpreted as the surface area of the solvent–polymer interface. Specifically, SASA is measured as the area circum-scribed by the motion of the center of a spherical probe that slides along the periphery of the polymer beads that constitute a cluster.^88^

### Information Entropy (*H*)

To quantify the changes in the configurational entropy associated with the packing of copolymers within various intermediate assemblies leading to vesiculation, we calculate the probability distribution function (pdf) of the extension of the polymer chains. First, the end-to-end distance of each chain (*Q*) in the cluster is calculated. Given *p*(*Q*), we calculate the information entropy, *H*, of the structure using its standard definition as *H*(*Q*) ≡ ∑ *p*(*Q*) ln (*p*(*Q*)),^89^ where the discrete summation is carried out for the ensemble of copolymers within a cluster. The end-to-end vector is the most widely used structural parameter in polymer kinetic theory.^90^ Specifically, *p*(*Q*) quantifies the distribution of polymer stretch, which depends on the polymer configuration. A broader *Q* distribution points to a greater configurational diversity and hence larger degrees of configurational freedom (entropy).

### Pair Correlation Function (*g*(*r*))

The pair correlation function (*g*(*r*)) describes how the density of B, A, or W beads varies as a function of distance from the center of mass of a cluster along the radial direction. A larger value of *g* signifies more tightly packed beads. In general, *g* shows (local density)/(overall density). *g*(*r*) is represented by
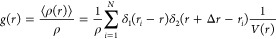
1where ⟨ρ(*r*)⟩ is the average density of beads at a distance, *r*, from the center of mass; ρ = 3*N*_*m*_/(4π*r*_max_^3^) is the average cluster density; *N*_*m*_ is the total number of monomer beads contained in the cluster; *r*_max_ is the distance of the farthest bead from the center of mass of the cluster; *V*(*r*) = (4/3)π[(*r* + Δ*r*)^3^ – *r*^3^]; and
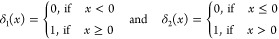
2

Equation 2 implies that the beads at the inner boundary are included, while those at the outer boundary are excluded. In the calculation, we used Δ*r* = 0.1 nm.

#### Ellipticity (*E*) and Vesicle Orientation (θ_*V*_, φ_*V*_)

In order to quantify flow-induced deformation and orientation of the vesicle, we calculate the radius of gyration tensor **R**_G_ of the structure. The eigenvalues (λ_1_ ≥ λ_2_ ≥ λ_3_) and corresponding eigenvectors (principal axes) of **R**_G_ are calculated. Ellipticity *E*≡ (λ_1_ – λ_3_)/(λ_1_ + λ_3_), is used to quantify the deviation of the vesicle shape from a spherical one for which *E* = 0. The eigenvector corresponding to the largest eigenvalue is used to calculate the vesicle orientation. The orientation angle θ_V_ is the angle between the projection of the eigenvector corresponding to λ_1_ onto the *x–y* plane and the *x*-axis. Similarly, φ_*V*_ is the angle between the projection of the vector onto the *x*–*z* plane and the *z*-axis. These angles are used to detect the flow alignment of the deformed vesicle.

## Results and Discussion

### Shape Deformation and Vesicle Rupture

**Figure 2:**
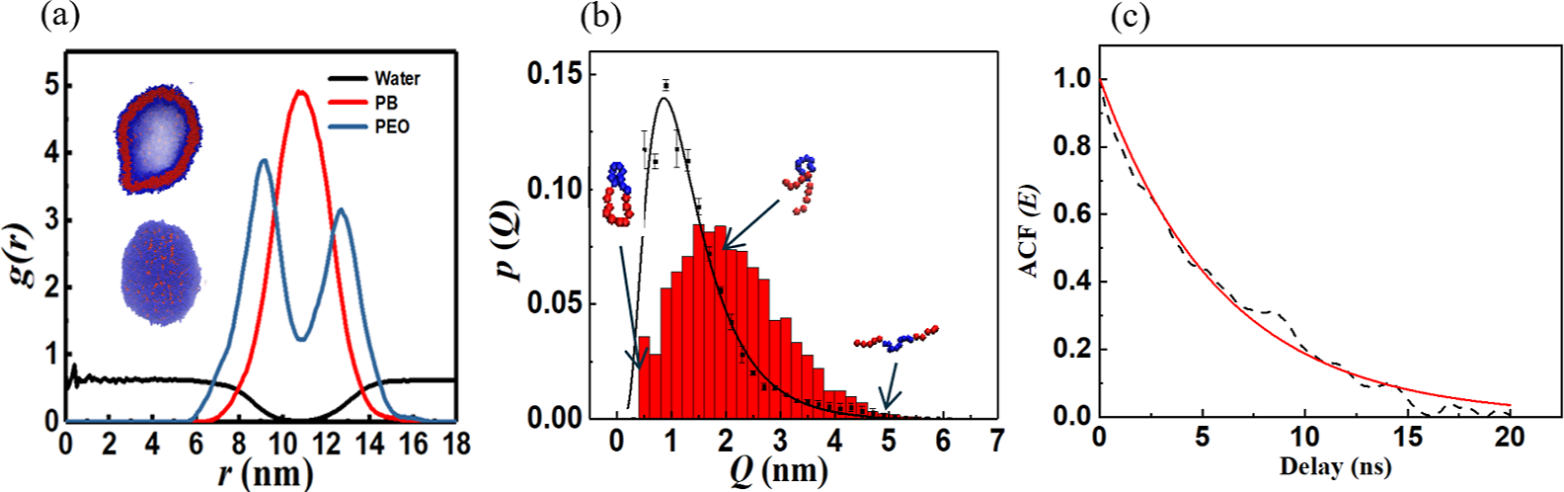
(a) Pair correlation functions of the PB, PEO, and water beads in the vesicle membrane. The insets show the full and a cross-sectional view of the vesicle. (b) Probability distribution functions of the end-to-end distance *Q* of the copolymer chains within the vesicle (red bars) and a single copolymer in solution (black curve). (c) Autocorrelation function (ACF) associated with the fluctuations in the ellipticity *E* of the vesicle at equilibrium (dashed line) and an exponential fit to the data (solid red line).

The pair correlation functions of the PB_5_–PEO_10_-PB_5_ vesicle at equilibrium are shown in Figure 2a which demonstrates that the self-assembled structure has a diameter of approximately 32 nm, a water core, and a well-defined PEO–PB-PEO bilayer ≈10 nm thick. A typical snapshot of the vesicle is shown in the inset of Figure 2a. The primary driving force underlying the vesiculation process is the unfavorable hydrophobic interactions between the PB segments and solvent.^30^ The pathway of vesiculation starting from a homogeneous solution of copolymer chains consists of the rapid formation of interconnected spherical aggregates, the merger of spherical aggregates to form a cage-like assembly made up of cylindrical micelles which reorganize into lamellar (bilayer) structures, and the closure of the lamellar cage to form a closed bilayer.^30^Figure 2b shows the probability distribution functions (pdfs) of the end-to-end distance *Q* of an isolated single copolymer chain in solvent, calculated from the time series data predicted by the equilibrium CGMD simulations of a single PB_5_–PEO_10_-PB_5_ chain in water and the copolymer chains within the vesicle. The pdf of *Q* for a single chain is the log–normal. The predominant configurations correspond to folded/hairpin-like shapes. Relatively less probable quasilinear shapes constitute the tail of the pdf.

The *p*(*Q*) for the vesicle is also non-Gaussian. However, the hairpin-like configurations are relatively wider, i.e., the end beads of the chain are pulled farther apart due to favorable interactions with the neighboring chains. Consequently, relative to the single chain, *p*(*Q*) is broader and shifted to larger *Q* values.

A suitably defined Weissenberg number, , where τ denotes a characteristic structure relaxation time, is traditionally used to qualitatively assess the influence of an imposed shear flow on structure deformation. Since *Wi* thus defined signifies the ratio of the time scale of an intrinsic relaxation process of the structure to that of the shear flow, significant morphology distortion can be expected for *O*(1) values of *Wi*. In this study, we use the time scale τ associated with the shape fluctuations of the vesicle at equilibrium in the definition of the Weissenberg number. Specifically, τ is defined as the time constant associated with the exponential decay of the temporal autocorrelation function (ACF) of the ellipticity of the vesicle: see Figure 2c. The relaxation time τ obtained by fitting the ACF to a single exponential function is 5.96 ± 0.04 ns. We hence use τ = 6 ns in calculating *Wi*.

**Figure 3:**
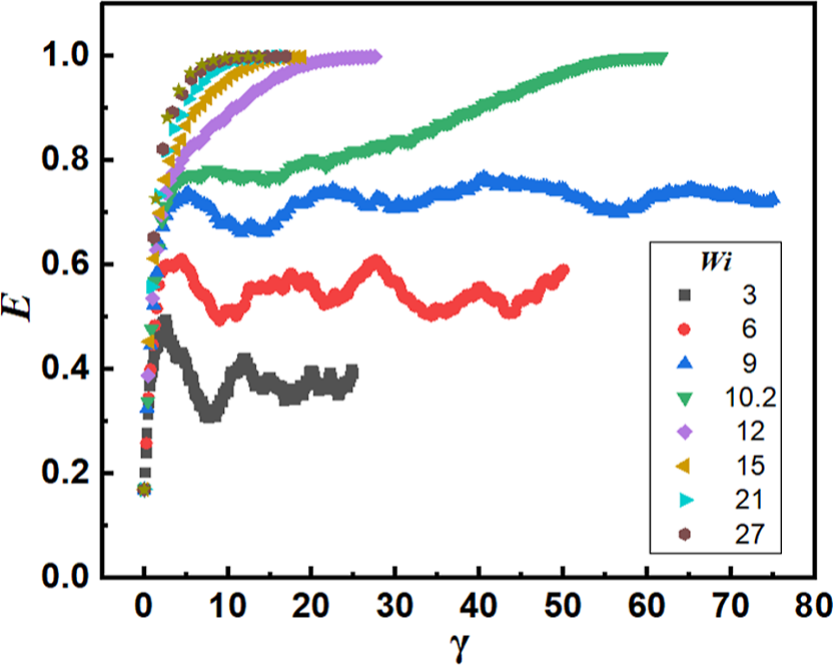
Variation in ellipticity *E* as a function of applied strain γ for different *Wi*. For *Wi* ≤ 9, vesicle rupture is not observed during the time span (50 ns) of the simulations. For *Wi* ≥ 10.2, the last datum shown corresponds to the time of vesicle rupture.

**Figure 4:**
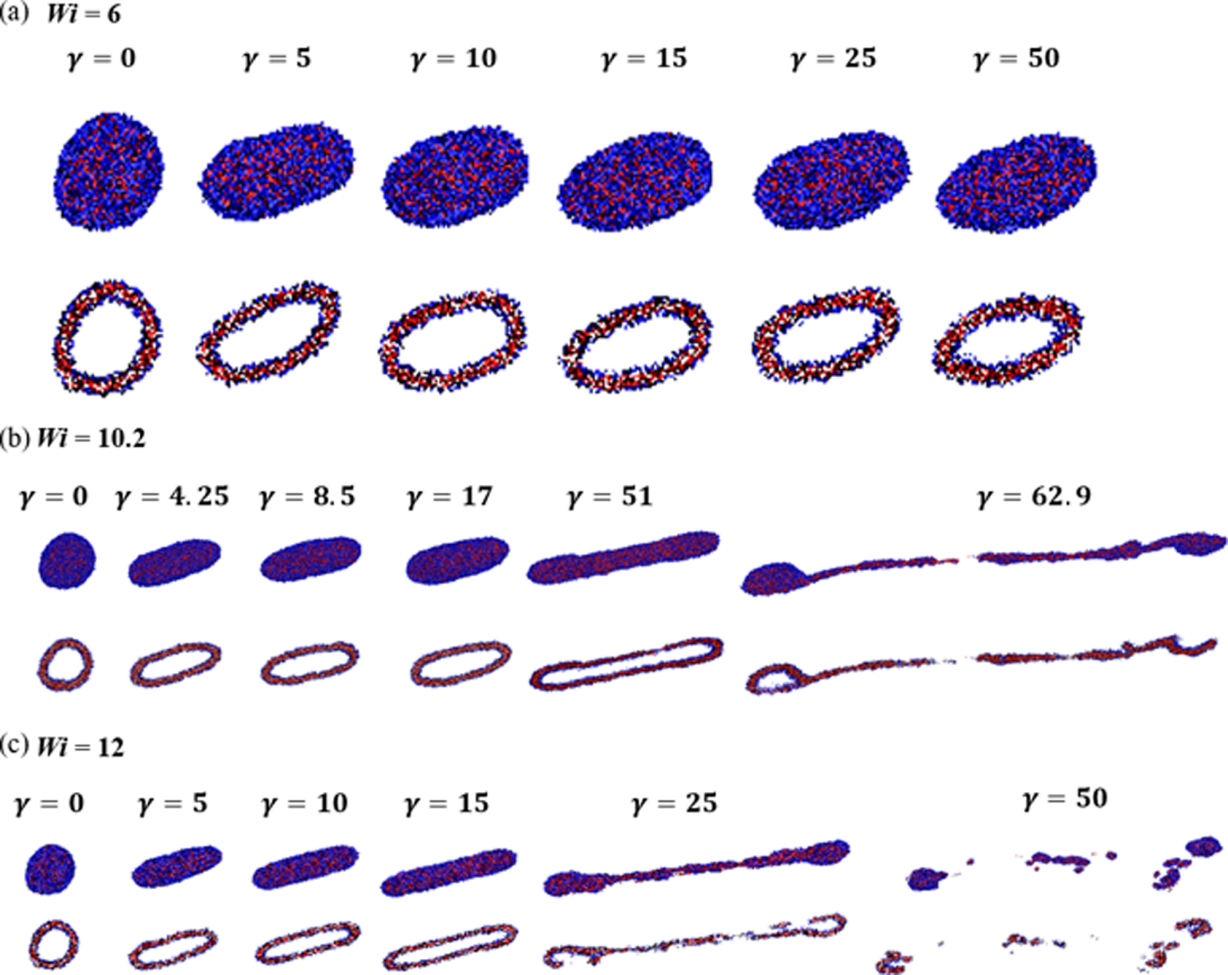
Front and cross-sectional views of the structure for (a) *Wi* = 6, (b) *Wi* = 10.2, and (c) *Wi* = 12 at different strains.

**Figure 5:**
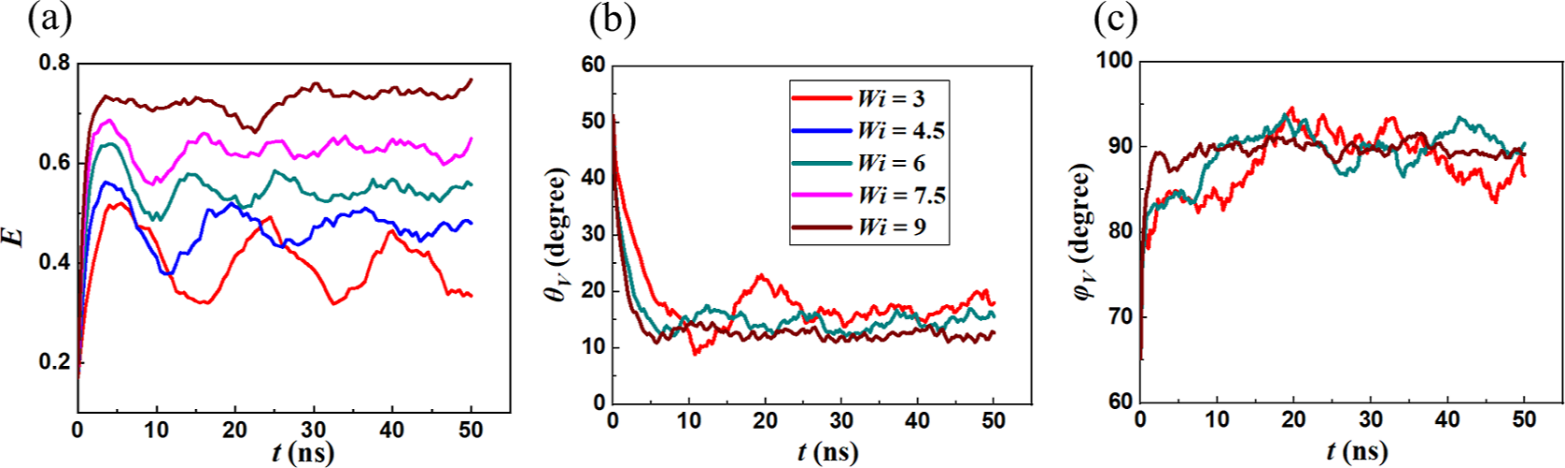
(a) *E*, (b) θ_*V*_, and (c) ϕ_*V*_ as functions of time for different *Wi*. Vesicle is flow-aligned and its major axis lies within the flow-shear plane.

Variations in *E* as a function of shear strain γ ≡ γ̇*t* for 3 ≤ *Wi* ≤ 27 are shown in Figure 3. For *Wi* ≤ 9, vesicle rupture is not observed during the time span of the simulations. In this flow regime, *E* increases monotonically as a function of γ during the application of the first few units of strain, signifying rapid vesicle deformation. Further, the stretched vesicle is seen to relax from a deformed state and undergo relatively small oscillations in *E*: see Figure 4a for snapshots illustrating the vesicle shape and orientation for *Wi* = 6 and 0 ≤ γ ≤ 50. The dynamics of *E*, as well as the orientation angles θ_*V*_ and φ_*V*_ of the major axis, are plotted in Figure 5. The major axis of the vesicle is seen to lie within the flow-shear (*x*–*y*) plane (φ_*V*_ ≈ 90°) subtending a shallow angle with the flow direction. The asymptotic behavior of the vesicle resembles the tank-treading motion observed experimentally for macroscopic lipid vesicles in shear flow.^52,57,60,91^ To analyze this similarity further, we computed volume *V* and surface area *A* of the vesicle during the deformation process and found that both quantities remain practically constant for *t* > 25 ns: for example, *V* = 9506 ± 84 nm^3^ and *A* = 3447 ± 187 nm^2^ for *Wi* = 9 (From *t* = 0 to 25 ns, *V* and *A* increases by 1% and 13%, respectively). For this case, the effective volume parameter ν ≡ 3*V*/(4π*R*^3^) where *R* =  asymptotes to 0.50 ± 0.04 while θ = 12.2° ± 0.7°. This is consistent with the θ_*V*_ values reported in previous studies of macroscopic lipid vesicles: Kraus et al.^91^ predicted θ ≈ 10° for the case in which the viscosities of the vesicle membrane and surrounding fluid are equal. Note that in the tank-treading regime, θ_*V*_ is insensitive to the dimensionless shear rate χ ≡ , where μ and κ denote the viscosity and bending modulus of the membrane. For μ = 10^–3^ Pa s and a typical *κ* value of 10^–19^ J, χ for the nanovesicle considered in this study is *O*(10).

For *Wi* ≥ 10.2, for large strains, the vesicle continues to deform and eventually ruptures. This behavior is illustrated in Figure 4b,c for *Wi* = 10.2 and 12, respectively. For *Wi* = 10.2, which lies close to the borderline of the tank treading and flow-induced rupture regimes, there exists a time period where the cohesive forces within the self-assembled structure resist rupture: see the region 4.25 ≤ γ ≤ 16.15, *E* ≈ 0.77 in Figure 3. However, the continued application of shear bootstraps the deformation process, leading to vesicle rupture: see Figure 4b for snapshots. For *Wi* = 12, the vesicle undergoes rapid deformation with *E* approaching its maximum value of unity prior to breakup: see Figure 4c for snapshots. The γ value at which *E* → 1 decreases with increasing *Wi*. For the largest *Wi* shown in Figure 3, *E* ≈ 1 for γ = 6.

**Figure 6:**
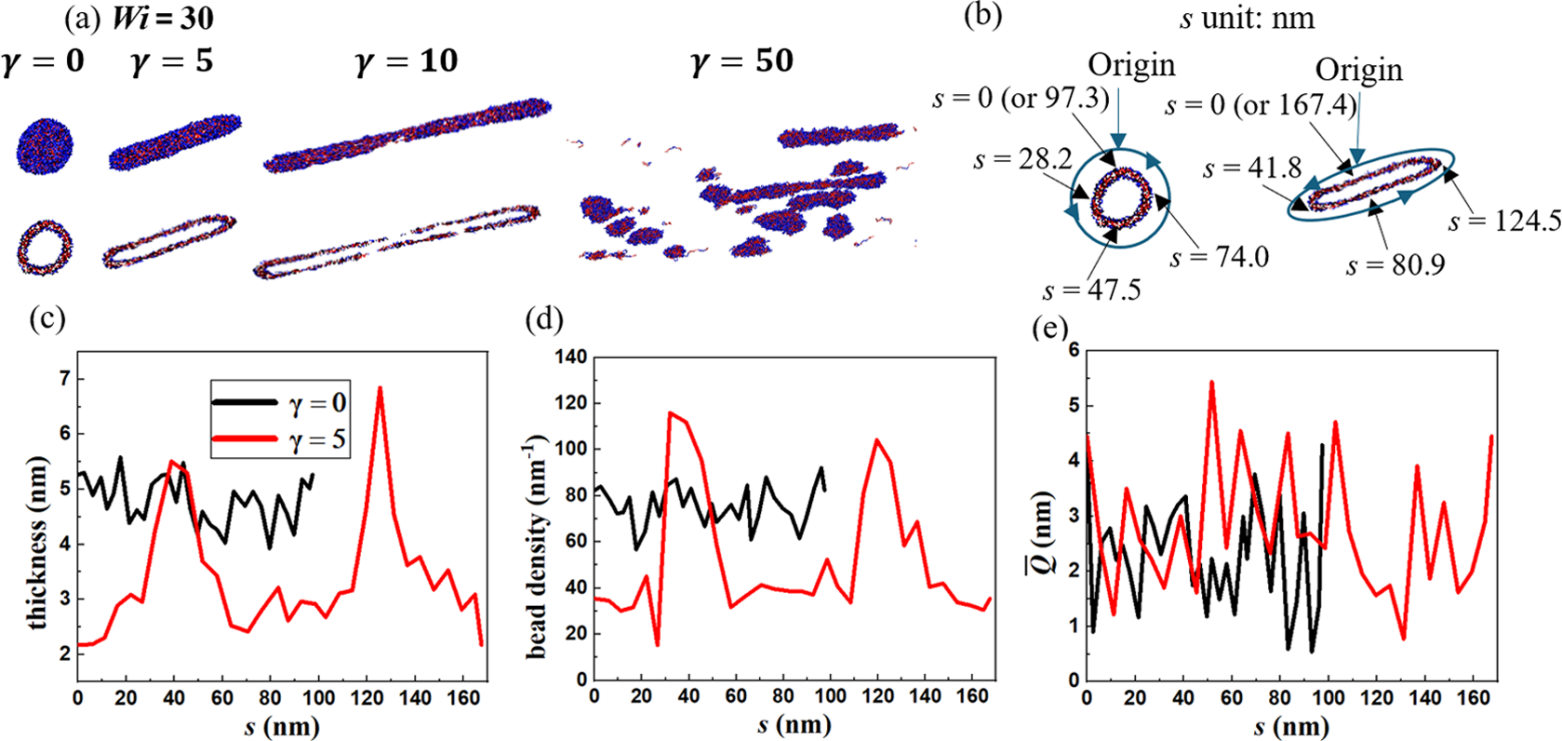
(a) Front and cross-sectional views of the vesicle for *Wi* = 30 at different strains; (b) schematic illustrating the contour distance variable *s*; (c) vesicle thickness, (d) bead density, and (e) *Q̅* vs *s*.

Snapshots illustrating vesicle deformation and rupture for *Wi* = 30 are shown in Figure 6a. To quantify the variations in bilayer thickness and polymer stretch during the deformation process, we employ the contour distance variable *s* illustrated in the schematic shown in Figure 6b. Specifically, *s* is defined with the midplane of the upper portion of the bilayer as its origin and increases as the vesicle is circumnavigated in the counterclockwise direction. As the vesicle stretches, copolymers are advected from its middle regions toward its ends. This results in an ellipsoidal vesicle with nonuniform thickness, i.e., thicker at the ends and thinner in the middle. Bilayer thickness and bead density (number of monomer beads per nm along the vesicle contour) as functions of *s* are shown in Figure 6c,d, respectively, for the equilibrium (γ = 0) and a deformed (γ = 5) vesicle. The vesicle at equilibrium has a nearly uniform wall thickness of 4.8 ± 0.4 nm and a constant bead density of 76 ± 8 beads/nm. In comparison, the thickness and bead density of the deformed vesicle exhibit two peaks at both ends. Figure 6e shows the average polymer end-to-end distance *Q̅* as a function of *s* for γ = 0 and 5 illustrating that the thinning of the midsection of the bilayer is accompanied by the unfolding and stretching of the copolymer chains in that region.

**Figure 7:**
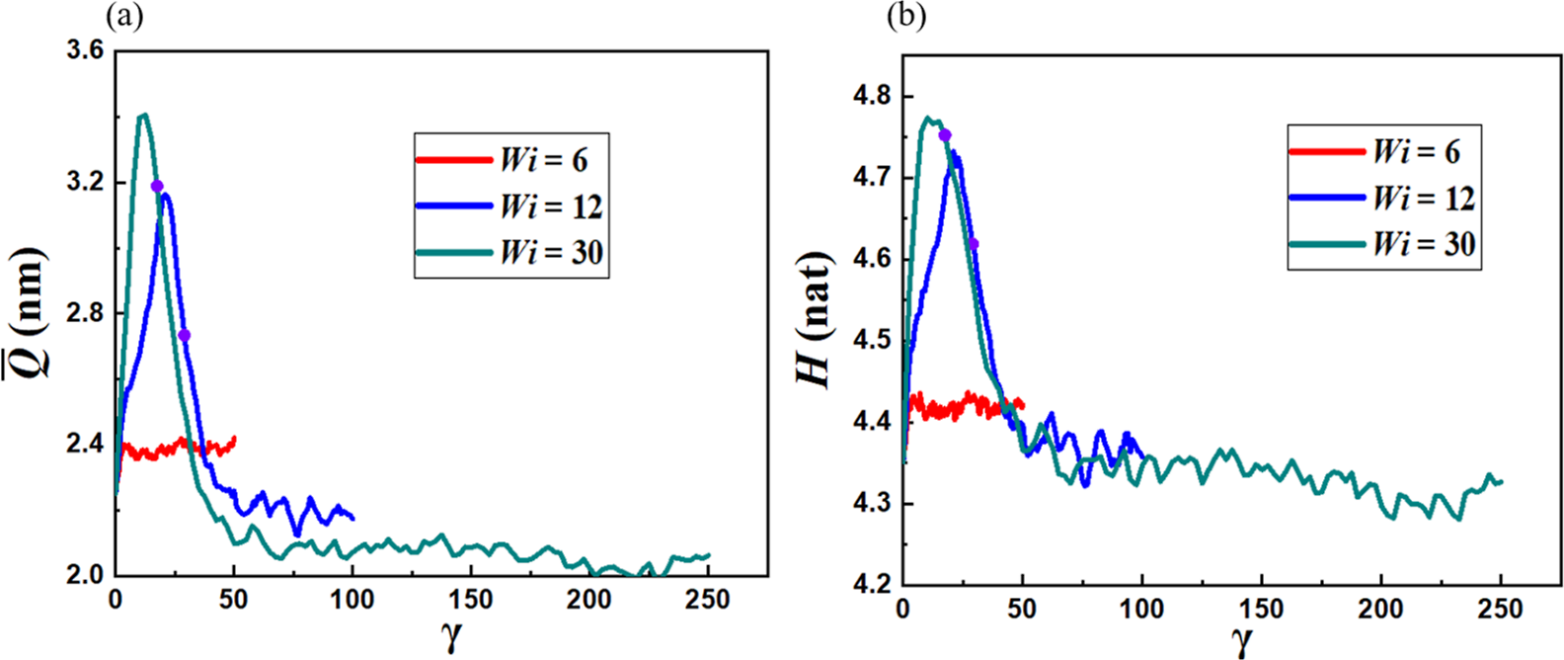
(a) Average chain end-to-end distance *Q̅* and (b) configurational entropy *H*. The purple dots indicate the strains corresponding to vesicle rupture.

For the two cases in which vesicle rupture is reported (*Wi* = 12, 30), the *Q̅* shown in Figure 7a increases rapidly with increasing strain and reaches its maximum at γ = 29.5 (*Wi* = 12) and 1.25 (*Wi* = 30), respectively. During this period, the copolymer chains in the middle regions unfold from a hairpin-like to a to a stretched, quasilinear configuration. Simultaneously, advection of copolymers depletes the middle part of the bilayer, resulting in defects that form randomly on the vesicle surface. These defects grow into larger holes, causing the vesicle to break up into fragments. The number and average size of the fragments increase and decrease, respectively, with increasing *Wi*. Stretched polymers within the fragments relax swiftly toward their equilibrium configurations, as indicated by the rapid decrease following rupture in *Q̅*. The information entropy *H* shown in Figure 7b also exhibits a rapid increase prior to breakup and decreases quickly to its equilibrium value after vesicle fragmentation. For *Wi* = 12, the high-density bilayer portions at both ends retain the water core and form smaller vesicles. The middle fragments lose their water core and reorganize into rods and spherical micelles. For *Wi* = 30, the elongated vesicle is broken into rods and spherical micelles. Once the vesicle ruptures, recombination and separation of copolymer aggregates characterize the system dynamics.

**Figure 8:**
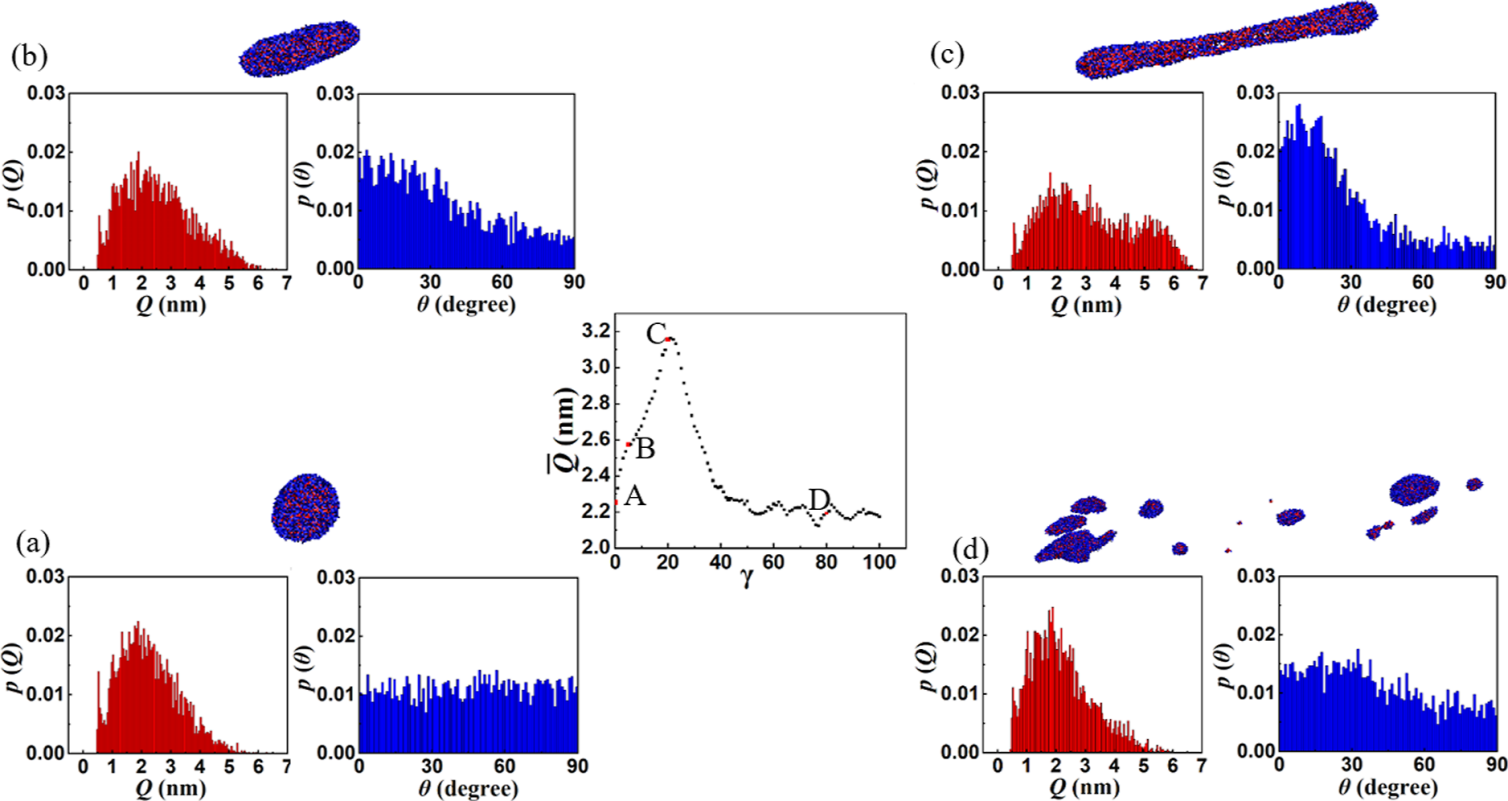
Average end-to-end distance *Q̅* vs strain γ for *Wi* = 12 (center panel). (a–d) Pdfs of *Q* and θ corresponding to the red dots A (equilibrium), B, C, and D (after rupture), respectively, are shown in the *Q̅* vs γ plot.

The increase in *H* accompanying vesicle deformation is caused by a broadening of *p*(*Q*), see Figure 8, which shows *Q̅* as a function of γ for *Wi* = 12 (reproduced from Figure 7a). The pdfs of *Q* and θ of the copolymer chains are also shown along with snapshots of the morphology for four points along the deformation path. The first point A corresponds to the equilibrium state in which the vesicle is nearly spherical. For this case, *p*(*Q*) follows a log-normal distribution, and *p*(θ) is uniform, signifying the absence of any preferred orientation. As the spherical vesicle is deformed into an ellipsoid, *p*(*Q*) shifts to larger values of *Q* and *p*(θ) develops a maximum at θ = 0 indicating stretching and flow-alignment of a significant portion of the chains within the bilayer. The highly deformed, elongated dumbbell shape at point *C*, corresponding to the maximum in the *Q̅* vs γ plot is characterized by a bimodal distribution of *Q*. This is consistent with the presence of stretched copolymers in the middle portion and folded chains at the ends of the dumbbell-shaped bilayer. The maximum in *p*(θ) is observed at ≈10° with the flow direction. This is close to the average orientation of the vesicle. After bilayer rupture, the copolymer chains relax, and the second peak in *p*(*Q*) seen in the dumbbell-shaped bilayer disappears. Further, since the chain orientation within the smaller fragments is unaffected by the shear flow, *p*(θ) distribution becomes more uniform.

**Figure 9:**
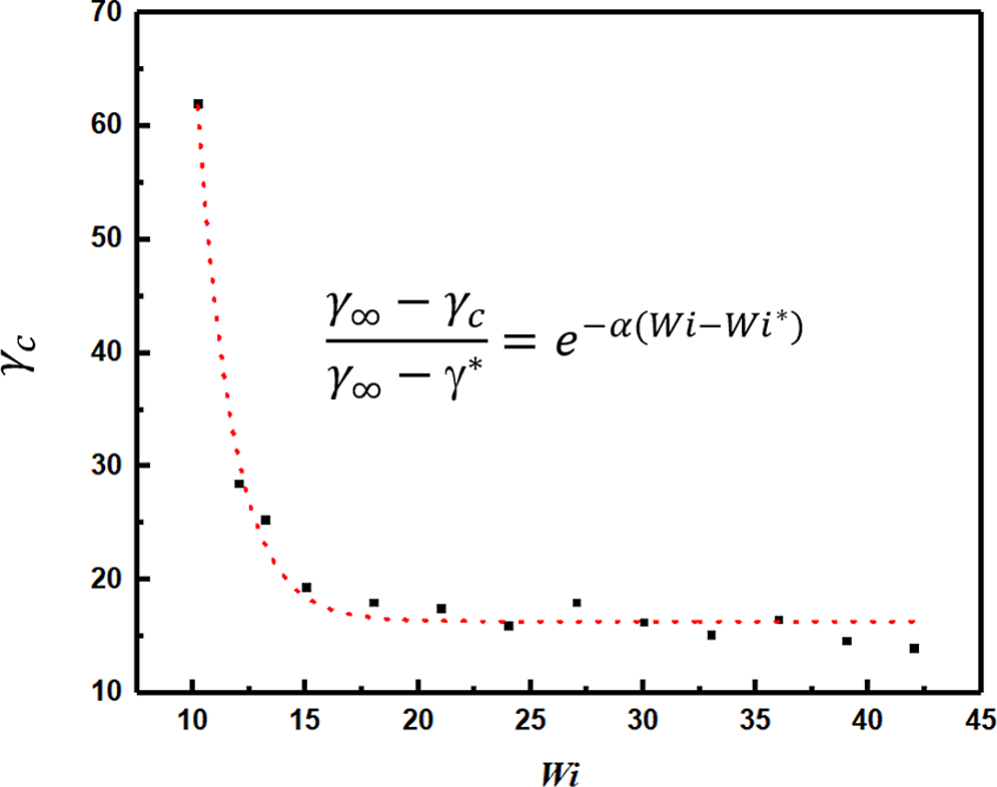
Critical strain γ_c_ of vesicle rupture as a function of *Wi*. γ* = 62 corresponds to the critical strain for the lowest Weissenberg number Wi* (= 10.2) for which vesicle rupture is observed., α = 0.548, γ_∞_ = 8.52.

Figure 9 shows the critical strain γ_c_ required to cause bilayer breakup as a function of *Wi*. The simulations suggest that both the shear rate and the shear strain need to be sufficiently large to facilitate vesicle rupture. The threshold Weissenberg number suggested by the simulations is ≈10.2, below which the tank-treading motion shown in Figure 5 is observed. As the shear rate (*Wi*) is increased above its critical threshold, the strain required to cause vesicle breakup decreases rapidly. The overall trend in the simulation data for γ* vs *Wi* can be described by an exponential fit given by , where the fitting parameters α = 0.548 and γ_∞_ = 8.52 with Wi* = 10.2 and γ* = γ (*Wi* = Wi*) = 62.

### Flow Cessation and a NoESIS

**Figure 10:**
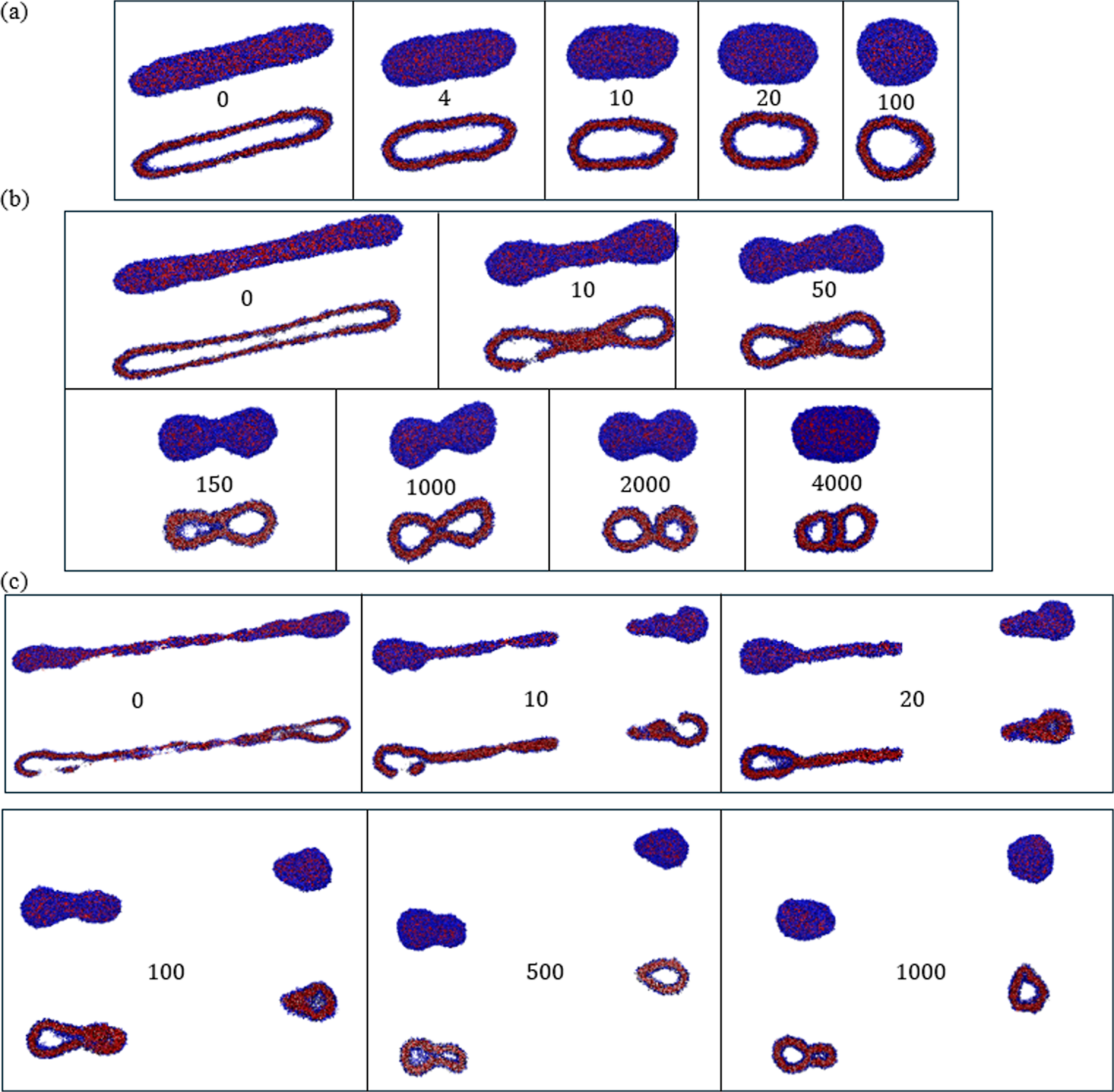
Front and cross-sectional views of the vesicle for *Wi* = 12. Cases shown correspond to flow cessation after a flow strain of (a) 15 (multimedia available online), (b) 20 (multimedia available online), and (c) 25 (multimedia available online). The time (ns) elapsed after flow cessation is indicated on each panel.

Relaxation of the deformed structures upon flow cessation was explored for a range of imposed strains for *Wi* = 12. As illustrated in Figure 10, three distinct behaviors were observed in the simulations depending on the maximum imposed strain γ_max_: see Videos S1–S3 provided in the Supporting Information. For γ_max_ = 15, the deformed ellipsoidal vesicle returns to its equilibrium spherical morphology (Figure 10a). However, when γ_max_ is increased to 20, the deformed vesicle assumes an elongated dumbbell shape with a bilayer that is nonuniform with regards to its thickness and copolymer extension. Figure 10b shows that the system preserves the shape memory of the *shear-induced structure* during the equilibration process. The final energy-minimized morphology, termed a *NoESIS*, corresponds to a composite copolymer assembly in which two distinct vesicular entities are connected by a dynamic molecular bridge.

For γ_max_ = 25, the applied strain greatly diminishes the structural integrity of the bilayer, which is at its early stages of breakup (Figure 10c). Equilibration from this state is accompanied by the breakup of the structure into two fragments. One of these aggregates evolves into a vesicular structure connected to a long lamella, which folds into itself to form another enclosed domain of solvent. Such lamella to vesicle reorganization is thermodynamically favorable since it allows for the shielding of the hydrophobic segments of the copolymers from water.^30,31,92^ The interface separating the two vesicular regions in this case consists of a bilayer of copolymers in their equilibrium (folded) configuration. In this aspect, this structure is similar to the bivesicle morphology that could result from the self-assembly of BAB triblock copolymers in aqueous solution under equilibrium conditions observed in our previous CGMD simulations.^32^ The second fragment consists of a vesicle attached to a micellar region, which retracts and merges into the former. Reorganization over time of this structure leads to the formation of a well-defined vesicle.

**Figure 11:**
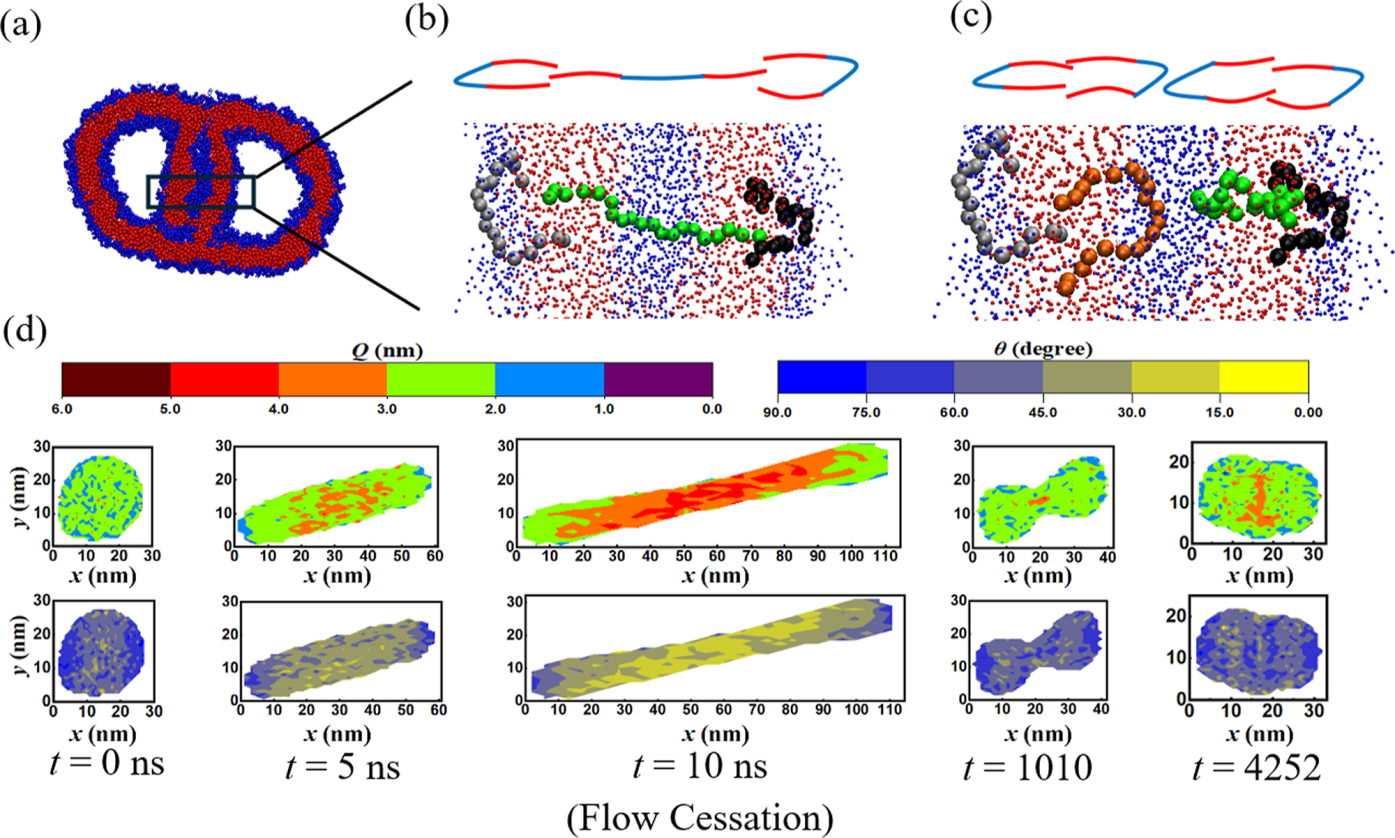
(a–c). NoESIS and the copolymer configurations within the interface connecting the two vesicular domains. Three-layer structures in which a stretched chain interpenetrates two folded chains (b) and quadruple layers consisting of four folded chains (c) are shown. Schematics of representative configurations are shown (top) for each case along with typical chain configurations obtained from the CGMD simulations. The red and blue dots represent PB and PEO monomers, respectively. (d) Distributions of chain end-to-end distance *Q* and angular orientation θ during shear and flow cessation for γ = 20, *Wi* = 12.

A detailed morphological analysis of the molecular architecture of the NOESIS formed at γ_max_ = 20 revealed that the interface connecting the two vesicular regions consists of two distinct types of copolymer organization, as illustrated in Figure 11. Specifically, the shared interface is not a bilayer. It consists of *three-layer* structures in which a stretched chain interpenetrates two folded chains (Figure 11b) and quadruple layers formed by two stacked bilayers of folded chains (Figure 11c). Hence, the NoESIS is structurally different from multicompartment vesicles (MCVs) synthesized under equilibrium conditions by the fusion or division of unilamellar vesicles.^49,93−95^ In MCVs, vesicular regions are typically separated by a classical bilayer of amphiphilic molecules. Figure 11d illustrates the retention of the memories associated with the shear-induced distortion in both the gross morphology and molecular configurations within the NoESIS: the elongated dumbbell shape of the vesicle prior to flow cessation (*t* = 10 ns) is preserved over a time span of over 100 times the equilibrium shape relaxation time of the parent vesicle. Second, the contour plots suggest the existence of a small fraction of highly stretched copolymers in the neck region of the dumbbell. These chains also retain their flow-alignment as demonstrated by the small region with θ < 15° within the interface (*t* = 4252 ns). However, configurations and positions of the center of mass of the copolymer chains within the layers of the molecular bridge are highly dynamic: the chains are observed to move within the bridge by a reptation-like mechanism, as illustrated in Video S4 provided in the Supporting Information.

**Figure 12:**
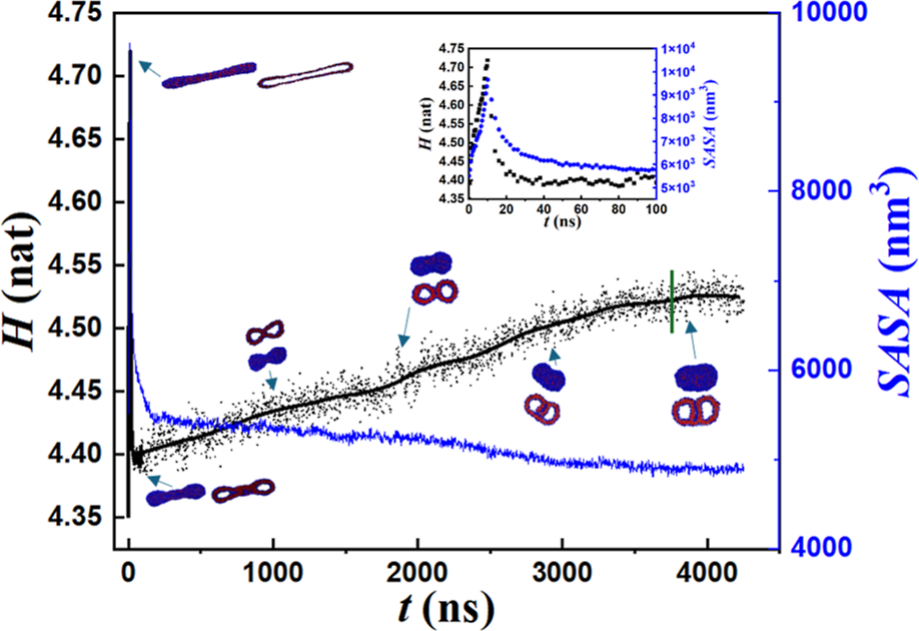
Variations in *H* (black dots) and *SASA* (blue line) along with snapshots of morphology during shearing and flow cessation for γ = 20 (*t* = 10 ns), *Wi* = 12. A magnified view for *t* < 100 ns is shown in the inset. The black solid line represents the smoothed line for *H* by the Lowess method.

We further analyzed the variations in *H* and *SASA* accompanying the flow–relaxation process that leads to the formation of the NoESIS. As shown in Figure 12, *SASA* increases rapidly with the increase in the interfacial area created by flow-induced morphology deformation. After flow cessation, the surface area decreases monotonically, and for long times, approaches a value ≈10% below its initial one. *H* also shows a rapid increase with shear-induced stretching of the copolymers. Flow stoppage causes a quick decay in *H* as the stretched chains relax up to *t* ≈ 150 ns. Beyond this stage, reorganization in morphology and copolymer configurations causes *H* to increase and asymptote to a value ≈4% above its initial one. This increase may be attributed to the presence of stretched polymers within the intervesicle interface as discussed above.

**Figure 13:**
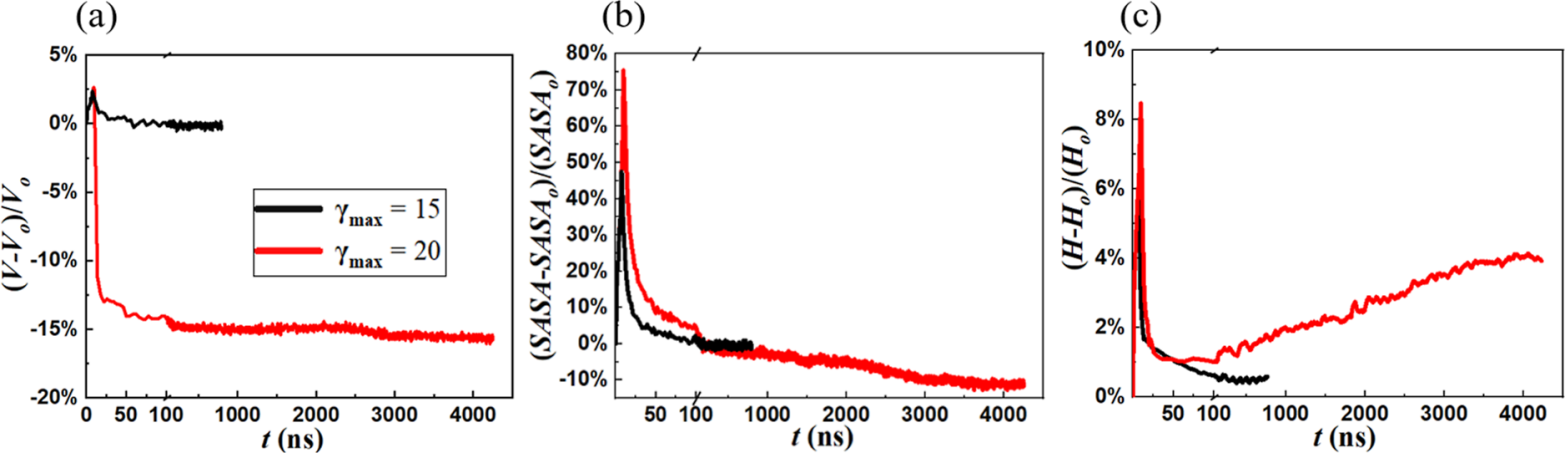
Percentage relative change in (a) vesicle volume *V*, (b) *SASA*, and (c) *H* for γ_max_ = 15 and 20.

In Figure 13, we compare the variations in the vesicle volume *V*, *H*, and *SASA* accompanying the flow startup–relaxation cycle for γ_max_ = 15, for which the vesicle returns to its original equilibrium, and γ_max_ = 20 for which the NoESIS is formed. *V* for the NoESIS is approximately 15% lower than that of its parent structure (unilamellar, spherical vesicle). The corresponding solvent–polymer interfacial area is nearly 10% lower in comparison. This can be attributed to the presence of additional polymer layers within the interface connecting the two vesicular domains of NoESIS. Polymers within these internal layers are shielded from the solvent. This suggests that reorganization of the deformed morphology after flow cessation follows an energetically favorable path by which a new morphology with a lower polymer–solvent interfacial area is created. Simultaneously, as discussed above and illustrated in Figure 13c, the configurational entropy for the NoESIS is approximately 4% greater than that of its parent structure due to the presence of stretched polymers within the intervesicle interface.

To assess the thermal stability of NoESIS reported in this work, we performed a CGMD simulation in which the temperature of the system corresponding to that shown in Figure 11d for *t* = 4452 ns was increased by 10 °C in a single step. This simulation did not reveal a substantive modification of the gross morphology by thermal perturbations even after 600 ns. Hence, the aforementioned energetic and entropic variations rendered by the flow startup–relaxation cycle facilitate the reorganization of the gross morphology and copolymer configurations of the parent structure into a new, stable equilibrium state.

## Conclusions and Perspective

CGMD simulations were conducted to study the dynamics of a unilamellar copolymer nanovesicle in startup and the cessation of uniform shear flow. The nanovesicle, formed by the spontaneous self-assembly of amphiphilic triblock copolymers under equilibrium conditions,^30^ has a bilayer membrane thickness comparable to its diameter. Further, the membrane consists of two stacked layers of mostly folded or hairpin-shaped copolymer chains. The simulations, which account for explicit solvent (water)-mediated interactions among the copolymer molecules, have the capability to track flow-induced modifications in the overall gross morphology, bilayer thickness, copolymer chain configurations, and the area of the polymer–solvent interface. By tracking the dynamics of these variables during the structure deformation–relaxation cycle, the simulations provide a comprehensive understanding of the molecular-level mechanisms of the flow-mediated reorganization of copolymer aggregates. Specifically, the simulations reveal hysteretic startup–cessation cycles of shear flow that open new corridors within the system’s thermodynamic topography, establishing morphological states seemingly inaccessible to equilibrium protocols.

For low to moderate flow strength, characterized by *Wi* < 10, nanovesicle dynamics can be described by the tank-treading motion observed in the case of large lipid vesicles in shear flow. Specifically, the deformed ellipsoidal vesicle remains within the flow–vorticity plane, with its longest axis subtending a shallow angle of approximately 10° with the flow direction. This is consistent with previous theoretical^57^ and computational^52,60^ predictions. For *Wi* > 10, shear strain distorts the vesicle into an elongated dumbbell shape. Advection of copolymers from the midregion of the vesicle to its ends creates pronounced nonuniformity in the bilayer thickness and produces a narrow neck near its center. The midsection of the dumbbell consists of copolymers stretched by flow shear, whereas copolymers at its ends exist predominantly in folded configurations resembling their organization within the equilibrium bilayer. Continued application of flow strain causes the neck region to rupture, giving rise to lamellar fragments and copolymer aggregates, which, by recombination and reorganization, could produce cylindrical micelles. For *Wi* > 10, the critical strain associated with vesicle rupture is seen to decrease rapidly with increasing shear rate, approaching a plateau for larger *Wi* (20 < *Wi* < 42).

Flow cessation studies show that shear-induced vesicle deformation is reversible below a critical γ_max_ ≈ 15 for *Wi* = 12. However, for γ_max_ above this threshold, hysteretic dynamics are observed in which the elongated dumbbell structure retains its shape and polymer configuration memory over time scales several orders of magnitude greater than the vesicle shape relaxation time at equilibrium. Consequently, structure reorganization during this period establishes a NoESIS in which two vesicular regions are connected by a molecular bridge whose composition is both highly heterogeneous and dynamic. Structure reorganization for γ_max_ values for which the bilayer ruptured after flow cessation, is accompanied by the creation of smaller nanovesicles.

An analysis of the variation in thermodynamic markers of the system during the flow start–stop cycle suggests that persistence and thermal stability of NoESIS may be attributed to an appreciable reduction in the polymer–solvent interface. This is achieved by shielding the hydrophobic segments within the multilayered molecular bridge from the solvent. Further, due to the presence of a small fraction of stretched polymers, the configurational entropy of the NoESIS is discernibly greater than that of its parent structure. To date, experimental observations of persistent or permanent shear-induced structure transitions in amphiphilic solutions^73−76^ have defied mechanistic explanations based on analyses of the effect of deformation history on thermodynamic motifs of self-assembly in well-defined model systems. The prospect of flow-induced morphological modifications driving an equilibrium molecular assembly into a kinetically trapped metastable state has been suggested as a broad-brush rationalization of such curious phenomena. Fine-grained simulations, such as the one performed in this work, are capable of simultaneously tracking individual molecular configurations, interfacial dynamics, and morphology deformation and thus capable of discovering flow-mediated pathways to the establishment of hitherto unknown molecular assemblies such as NoESIS. Further, studies focused on a comparative analysis of the hysteretic structures formed at different shear strains, quantitative determination of molecular motion within the equilibrium bilayer and NoESIS layers, as well as a thorough examination of the mechanical and thermal stability of NoESIS, will help deepen our understanding of flow-mediated pathways of amphiphilic assembly.

## Supporting Information Available

Flow startup and cessation, γ_max_ = 15 for *Wi* = 12 (MP4)
Flow startup and cessation, γ_max_ = 20 for *Wi* = 12 (MP4)
Flow startup and cessation, γ_max_ = 25 for *Wi* = 12 (MP4)
Copolymer chain motion within the NoESIS bilayer (MP4)
Simulation Details, parameter values for nonbonded interactions for the copolymer, parameter values for bond stretching interactions, and parameter values for bond bending interactions (PDF)

## Abbreviations

PB: polybutadiene;
PEO: poly(ethylene oxide);
NoESIS: Novel Equilibrated Shear-Induced Structure;
BCP: block copolymer;
SCFT: self-consistent field theory;
TEM: Transmission Electron Microscopy;
NMR: nuclear magnetic resonance;
CGMD: coarse-grained molecular dynamics;
NE-CGMD: nonequilibrium CGMD;
pdf: probability distribution function.
