# Supplementary material for: Deformation, Rupture, and Morphology Hysteresis of Copolymer Nanovesicles in Uniform Shear Flow
Source: Langmuir. 2024 Dec 31;41(8):5083–96. doi: 10.1021/acs.langmuir.4c04200 (PMC11887437; doi:10.1021/acs.langmuir.4c04200)
Supplement: Supplementary file 5 — la4c04200_si_005.pdf [file la4c04200_si_005.pdf]

Supporting Information

Deformation, Rupture and Morphology Hysteresis of Copolymer Nanovesicles in Uniform Shear Flow

Senyuan Liu [1], *and Radhakrishna Sureshkumar* [1,2,*]

[1] Department of Biomedical and Chemical Engineering and the Bioinspired Institute, Syracuse University, Syracuse, NY 13244, USA; sliu69@syr.edu

[2] Department of Physics, Syracuse University, Syracuse, NY 13244, USA

* Correspondence: rsureshk@syr.edu

Number of pages: 5

Number of figures: 0

Number of tables: 3

Table of Contents

Simulation Details: Pages 2-4

Table S1: Page 4

Table S2: Page 4

Table S3: Page 5

Simulation Details

The force field constraints of the molecular models used in this work were based on the requirements of the MARTINI force field[80].

The stretching and bending interactions between bonded beads were modeled by weak harmonic potentials V_b and V_θ, respectively, given by Equations (S1) and (S2) below:

$$V_b\,(b) = \frac{1}{2}K_b(b\ - b_0)^2 \quad , \tag{S1}$$

$$V_\theta\,(\theta) = \frac{1}{2}K_\theta(\cos{(\theta)}\ - \cos{(\theta_0)})^2 \quad , \tag{S2}$$

where b_0 and θ_0 represented the equilibrium bond distance and equilibrium bond angle at the minimum energy configuration, respectively, and K_b and K_θ represented constants associated with bond stretching and bending energies, respectively. The non-bonded interactions between beads i and j were described by a 6–12 Lennard−Jones (LJ) potential given by

$$V_{LJ}(r_{ij}) = \frac{C_{ij}^{(12)}}{r^{12}} - \frac{C_{ij}^{(6)}}{r^6} \quad . \tag{S3}$$

Equation (S3) can also be written as

$$V_{LJ}(r_{ij}) = 4\varepsilon_{ij}\left[\left(\frac{\sigma_{ij}}{r}\right)^{12} - \left(\frac{\sigma_{ij}}{r}\right)^{6}\right] \quad , \tag{S4}$$

here

$$C_{ij}^{(12)} = 4\varepsilon_{ij}\sigma_{ij}^{12} \ \text{ and } \ C_{ij}^{(6)} = 4\varepsilon_{ij}\sigma_{ij}^{6} \quad . \tag{S5}$$

In the above equations, r represents the distance between beads i and j, ε_{ij} is the depth (minimum) of the potential well (energy function), and σ_{ij} is the distance at which V_{LJ} is zero. ε_{ij} and σ_{ij} are also referred to as the interaction strength and cutoff length, respectively.

MARTINI force field requires the LJ potential to be shifted smoothly to 0 between 0.9 and 1.2 nm, rather than using a hard cutoff to avoid singularities in the computation of the inter-particle forces. This is achieved by using a potential−switch function S_V given by

$$S_V(r_{ij}) = C_{ij}^{(12)}S^{(12)} - C_{ij}^{(6)}S^{(6)} \quad , \tag{S6}$$

where

$$S^{(\alpha)} = \begin{cases} \frac{1}{r^\alpha} - C\ ;\ r \le r_{shift} \\ \frac{1}{r^\alpha} - \frac{A}{3}\left(r - r_{shift}\right)^3 - \frac{B}{4}\left(r - r_{shift}\right)^4 - C\ ;\ r_{shift} < r \le r_{cut} \\ 0;\ r > r_{cut} \end{cases} \quad . \tag{S7}$$

$$\alpha\ = 6\ or\ 12$$

In Equation (S7), the parameters A, B, and C are defined by

$$A = -\alpha\frac{(\alpha+4)r_{cut} - (\alpha+1)r_{shift}}{r_{cut}^{\alpha+2}\left(r_{cut} - r_{shift}\right)^2} \quad , \tag{S8}$$

$$B = \alpha \frac{(\alpha+3)r_{cut} - (\alpha+1)r_{shift}}{r_{cut}^{\alpha+2}(r_{cut} - r_{shift})^3} \quad , \tag{S9}$$

and

$$C = \frac{1}{r_{cut}^{\alpha}} - \frac{A}{3}\left(r_{cut} - r_{shift}\right)^3 - \frac{B}{4}\left(r_{cut} - r_{shift}\right)^4 \quad , \tag{S10}$$

where values of r_{shift} = 0.9 nm and r_{cut} = 1.2 nm are used. The force field parameter values used in this study are listed in Tables S1–S3. For computational efficiency, the same molar masses are assigned to each monomer and CG water (72 au) in equilibrium simulations. For consistency, the same masses are used in NEMD simulations. Figure 1(b) shows that the correct linear velocity profile corresponding to a uniform shear flow is recovered from the simulations. The MARTINI CG framework has been used in non-equilibrium simulations in the past for amphiphilic fluids yielding predictions of flow-induced structure modifications consistent with experimental observations[61,81-83].

In the present simulations, water (modeled as a four molecule cluster following the MARTINI prescription, represented by W), butadiene monomer (B) and ethylene oxide monomer (EO) are modeled by bead types P4, C4, SNda, respectively[80,84]. The solvent consists of 90.9% of P4 water beads along with 9.1% of "antifreeze" beads (represented by WF) which are of type BP4 and slightly bigger than the P4 beads. The WF beads are employed to disturb the lattice packing of the uniformly sized solvent beads and decrease the solvent freezing point[85]. The chemical affinities of bead types are described in the paper of Marrink *et al*[80].

The equilibrium simulations are performed by using the *Gromacs* 2020.2 MD software. First, simulations are conducted to minimize the energy of the initial system by the steepest descent algorithm. This is followed by a short (2 ns in the equilibrium simulations) NVT simulation to equilibrate the system at a desired temperature to ensure algorithmic stability. Subsequently, a sufficiently long NPT simulation (production run) is carried out (600 ns in the equilibrium simulations). The reason for conducting NVT simulation first is because that velocity generation at the outset of a simulation, which is done by sampling from a Gaussian distribution that yields the mean temperature, is imperfect. When coupled with a barostat, initial velocity distributions thus generated can frequently lead to numerical instabilities. Hence, equilibration is better performed for an NVT ensemble for a short period of time to get the correct the velocity distribution. Proceeding NPT simulations lead to the establishment of the appropriate system density.

The initial simulation box is cubic with a linear dimension of 40 nm. In comparison, the contour length of the longest copolymer chain is 6.64 nm. During the NPT simulation the box size changes to between 39 and 41 nm to match the correct system density.

The reference pressure and temperature are 1 bar and 300K, respectively. A v-rescale thermostat with a time constant of 1.0 ps is used for temperature coupling. Berendsen barostat with a time constant of 4.0 ps is used for isotropic pressure coupling during the NPT simulations. The simulations employ periodic boundary conditions along all three spatial coordinates. The time step for equilibration runs is 20 fs and the run time is 2 ns. The time step for production runs is 40 fs and the run time is up to 600 ns.

The shear flow (NE-CGMD) simulations are performed by using the *LAMMPS* 2 AUG 2023 MD software. The vesicle generated from the equilibrium simulations is placed into the solvent environment. To account for flow-induced vesicle stretching, the box dimensions in the NE-CGMD simulations

are increased to 200 nm × 80 nm × 40 nm. The additional volume thus created is filled with water and antifreeze water beads in 10:1 ratio. The vesicle generated from the equilibrium simulations is placed into this new solvent environment and the resulting system is equilibrated by a set of sufficiently long NPT simulations. The vesicle bead coordinates are fixed during the equilibrium process and only the W and WF beads coordinates are modified. The purpose of this process is to position the added solvent beads in reasonable positions without changing the vesicle structure. The overlaps and gaps that occur when the new solvent beads are randomly created are eliminated. Equilibration is achieved by a series of 5 NPT simulations in which the time-integration of the momentum equations is performed for one million steps each. The time step size is varied from 1 fs to 5fs in increments of 1 fs after every one million steps. This ensures the numerical stability of the simulations.

Thus, the shear simulation system has 4,000,240 water beads, 400,000 antifreeze water and 3871 PB_5-PEO_{10}-PB_5 copolymer chains. This corresponds to a dilute system with a copolymer concentration of 0.122 wt%. The NE-CGMD simulations are in NVT conditions.The reference temperature is 300 K. The SLLOD formulation of the Newton's equations of motion is used to generate desired uniform shear flows[86,87]. Periodic boundary conditions are applied along all three spatial coordinates. If a bead crosses the lower or upper boundary in the y direction, its x-velocity v_x is reset by adding or substracting $\overline{v_x}(L_y = L_{ymax}) - \overline{v_x}(L_y = L_{ymin})$, respectively. The time step used in the production runs is 5 fs and the simulation time is 50 ns.

Table S1. Parameter values for non-bonded interactions for the copolymer.

Interaction type	ε_{ij}(kJ/mol)	σ_{ij}(nm)
PB-W	2.7	0.47
PB-PB	3.5	0.47
PB-PEO	3.1	0.47
PEO-PEO	3.375	0.43
PEO-W	4	0.47
W-W	5	0.47
W-WF	5.6	0.57
WF-WF	5	0.47
PEO-WF	4	0.47
PB-WF	2.7	0.47

Table S2. Parameter values for bond stretching interactions.

Bond type	b_0(nm)	K_b(kJ/(mol · nm^2))
PB-PB	0.47	1250
PB-PEO	0.47	1250
PEO-PEO	0.33	17000

Table S3. Parameter values for bond bending interactions.

Angle type	θ_0 (degree)	K_θ (kJ/mol)
PB-PB-PB	180	25
PB-PB-PEO	180	25
PB-PEO-PEO	120	50
PEO-PEO-PEO	120	50
